# Progress of ketogenic diet in the treatment of developmental epileptic encephalopathy

**DOI:** 10.3389/fped.2025.1567095

**Published:** 2025-08-04

**Authors:** Wandong Hu, Lili Li, Fen Zhao, Huan Zhang, Hongwei Zhang

**Affiliations:** Department of Neurology, Children’s Hospital Affiliated to Shandong University, Jinan, Shandong, China

**Keywords:** ketogenic diet, developmental epileptic encephalopathy, refractory epilepsy, developmental delay, early-onset drug-refractory epilepsy

## Abstract

Developmental epileptic encephalopathy (DEE) is a severe neurological disorder caused by underlying genetic abnormalities and frequent epileptic activity. It is characterized by early-onset, drug-resistant epilepsy, abnormal electroencephalogram (EEG) findings, and developmental delay or regression. DEE is associated with high rates of disability and mortality. The ketogenic diet (KD) is a well-established non-pharmacological treatment for refractory epilepsy and has demonstrated therapeutic efficacy in several DEE subtypes. In certain cases, it may reduce or even eliminate the need for pharmacological interventions. This review discusses the current clinical application of KD in children with DEE and summarizes key factors influencing its therapeutic effectiveness.

## 1 Introduction

The term “developmental and epileptic encephalopathy” (DEE) was formally introduced by the International League Against Epilepsy (ILAE) in 2017 to describe a group of disorders resulting from a combination of genetic mutations and frequent epileptic seizures, both of which contribute to developmental impairments or regression in affected individuals (1). Importantly, this concept highlights that genetic abnormalities can independently cause developmental delays, which may be evident even before seizure onset. Frequent seizures subsequently exacerbate these developmental deficits (2, 3). DEE represents a particularly severe form of drug-resistant epilepsy, marked by early-onset, frequent seizures; developmental delay or regression; and cognitive and behavioral impairments. These conditions are associated with high rates of disability, increased mortality, and poor quality of life, posing significant challenges in pediatric neurology. In addition to pharmacological therapy, treatment options include neuromodulation, epilepsy surgery, and dietary interventions. Although epilepsy surgery can be effective in some focal epilepsies and neuromodulation may reduce seizure frequency in select cases (4, 5), many patients continue to experience refractory seizures. The ketogenic diet (KD) has gained recognition as an effective therapy for a range of seizure types, drug-resistant epilepsies, and specific metabolic disorders such as glucose transporter deficiencies (5–7). DEE encompasses a wide range of epilepsy syndromes, including infantile epileptic spasm syndrome [IS; also known as West syndrome (WS)], Ohtahara syndrome (OS), Dravet syndrome (DS), Lennox–Gastaut syndrome (LGS), epilepsy with myoclonic-atonic seizures (EMAS), continuous spike-and-wave during slow-wave sleep (CSWS), and Landau-Kleffner syndrome, as well as epilepsy associated with pathogenic mutations in genes such as *SCN1A*, *KCNQ2*, *STXBP1*, and *SCN2A*. Numerous studies have shown that KD is effective in managing these syndromes (7–9). Despite its efficacy and cost-effectiveness, KD remains underutilized in clinical practice. This review explores the current status of KD application in pediatric DEE and discusses the factors that influence its therapeutic outcomes.

## 2 Review methodology

This article is structured as a narrative review aiming to provide a comprehensive overview of the current progress in the use of the KD for the treatment of DEE. A literature search was performed in PubMed, Web of Science, and Embase databases using the following keywords: “ketogenic diet”, “epilepsy”, “developmental and epileptic encephalopathy”, “DEE”, “treatment”, and “mechanism”. The search included articles published between the inception of each database and January 2024.

Eligible studies included original research articles, clinical trials, systematic reviews, meta-analyses, and narrative reviews that focused on the therapeutic applications, mechanisms of action, and adverse effects of KD in patients with DEE. Articles that centered on epilepsy syndromes unrelated to DEE or non-KD interventions were excluded. The initial selection was based on screening of titles and abstracts, followed by full-text review to confirm relevance. Additional references were identified through citation tracking.

Due to the heterogeneity of study types and outcomes, this review presents a qualitative, narrative synthesis rather than a quantitative meta-analysis.

## 3 Types of KDs

The therapeutic potential of fasting has been recognized in epilepsy since the time of Hippocrates (460–370 BC), and the first scientific report describing its benefits in epilepsy was published in the 19th century by French physicians Guelpa and Marie, who observed reduced seizure severity during fasting (10). KD was first proposed by Wilder in 1921 and became widely used for epilepsy management by 1930, achieving remarkable results (11). KD is a high-fat, low-carbohydrate, protein-sufficient diet that remains a well-established and effective non-pharmacological treatment for intractable epilepsy in children (10). Currently, there are four main KD dietary approaches (7): (1) The classic KD (CKD), first described by Wilder in 1921 (11), derives 80%–90% of caloric intake from fats, primarily in the form of long-chain triglycerides, with a fat-to-carbohydrate-plus-protein ratio of 4:1. This is the most commonly used dietary regimen in infants and young children with DEE (12). For children with higher protein requirements, the ratio may be modified to 3.5:1 or 3:1, improving dietary acceptance and tolerability (13). CKD remains the most extensively studied and clinically effective form of KD (12, 14), especially in children under 2 years of age (15). (2) The modified Atkins diet (MAD), first reported in 2003, comprises approximately 60% fat, 30% protein, and 10% carbohydrates (16). It is a more palatable and less restrictive regimen that can be initiated in an outpatient setting (17). MAD is generally better tolerated and associated with fewer side effects, making it suitable for children, adolescents, and adults, particularly when CKD is not feasible due to behavioral issues or caregiver concerns (13). (3) The medium-chain triglyceride diet (MCTD), introduced in the 1950s, derives approximately 70% of total caloric intake from fat, with medium-chain triglycerides (MCTs) serving as the primary fat source and relatively low protein and carbohydrate intake. MCTs yield more ketone bodies per kilocalorie of energy than long-chain triglycerides, allowing for a reduction in total fat and an increase in permissible carbohydrates, thus expanding dietary variety (18). MCTD has shown comparable efficacy to CKD in controlling seizures and is more palatable and effective in children (19, 20). However, its broader use is limited by gastrointestinal side effects, including abdominal discomfort and bloating (21, 22). (4) The low glycemic index treatment (LGIT), introduced in 2005, is another dietary option for refractory epilepsy (23). LGIT was developed based on the importance of stable glucose levels observed in KD. The glycemic index quantifies the impact of foods on serum glucose levels (24). LGIT allows for a daily carbohydrate intake to 40–160 g, restricted to foods with a glycemic index below 50, such as meats, dairy products, certain fruits, and whole grain breads (23). While LGIT has comparable efficacy to CKD, its slower onset of ketogenesis in the initiation phase makes it less suitable for DEE and super-refractory status epilepticus.

## 4 Antiseizure mechanism of KD

**Figure 1:**
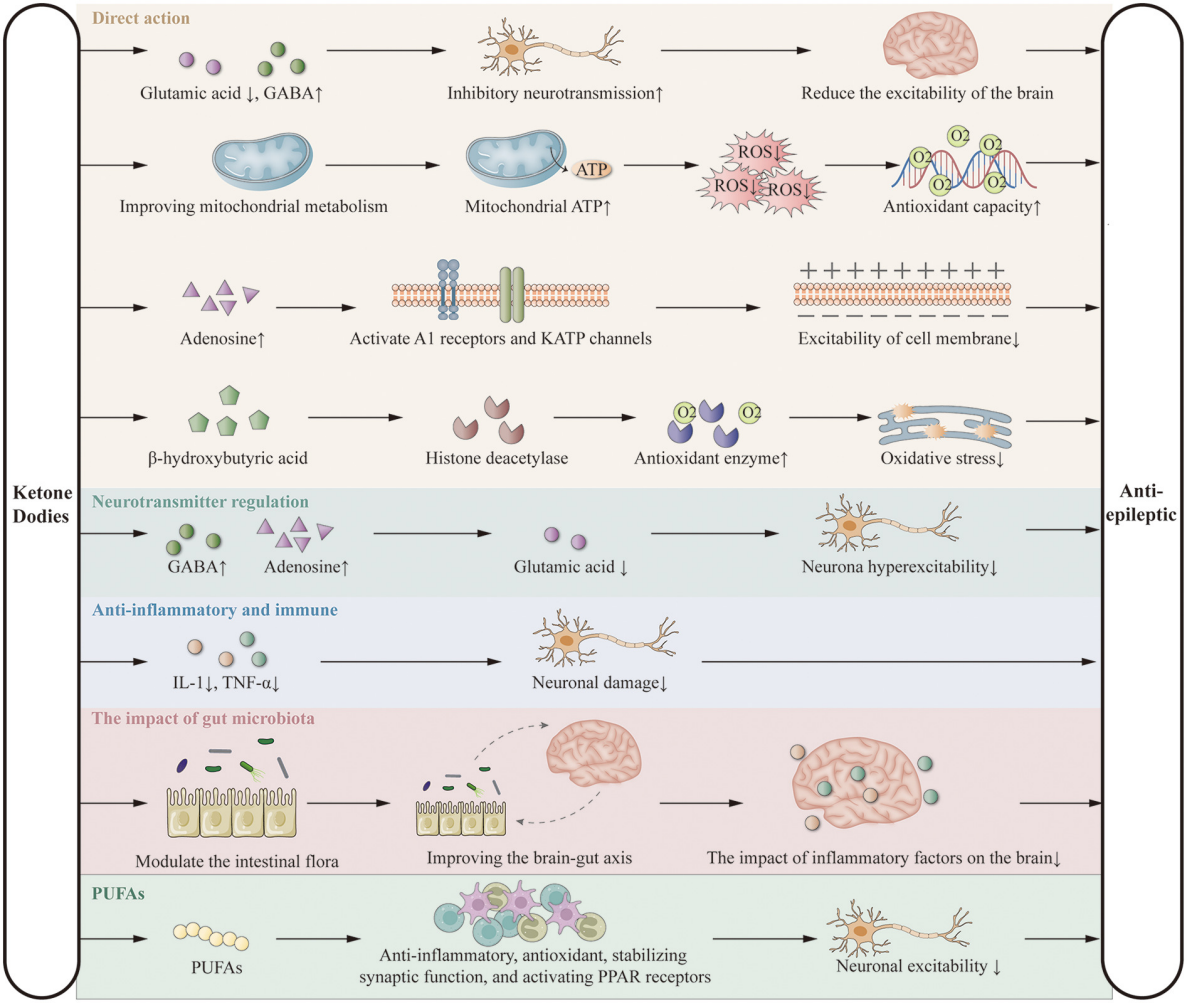
Putative anticonvulsant mechanisms of the ketogenic diet.

KD exerts antiepileptic effects primarily by limiting carbohydrate intake, inducing the production of ketone bodies (acetone, acetoacetate, and β-hydroxybutyrate), and utilizing them as alternative energy sources to glucose. These ketone bodies reduce neuronal excitability by decreasing glutamate release and enhancing GABA synthesis (25, 26), improve mitochondrial ATP production and antioxidant capacity (27, 28), increase adenosine levels via A1 receptor and KATP channel activation (29), and regulate gene expression through inhibition of histone deacetylases (30, 31). KD also modulates neurotransmitter balance (32–34), suppresses inflammation by reducing cytokines such as IL-1 and TNF-α (35–37), alters gut microbiota composition (38, 39), and exerts additional effects via polyunsaturated fatty acids (PUFAs) (13, 40, 41). These mechanisms form the theoretical basis for its potential benefits in DEE (Figure 1). Although the precise mechanisms are not yet fully elucidated, the clinical efficacy of KD is widely recognized. Further research is needed to explore these mechanisms in greater detail.

## 5 KD in the treatment of DEE

KD is a specialized dietary therapy widely recognized as effective for refractory epilepsy and DEE. DEE often has underlying genetic causes, typically involving single-gene or polygenic mutations. Current evidence consistently supports the efficacy of KD in treating DEE, with studies showing superior outcomes in certain epilepsy syndromes associated with genetic variations compared with pharmacological treatments (9, 42).

### 5.1 WS/IS

WS is a distinct epileptic syndrome characterized by clusters of spasms, hypsarrhythmia on EEG, developmental delay, and progressive neurological deterioration (43). First-line therapies typically include adrenocorticotropic hormone (ACTH) and vigabatrin; however, these are only partially effective and are associated with significant side effects (44). Clinical studies have demonstrated that KD successfully terminated seizures in 53.5% (23/43) of children and led to a >90% reduction in seizure frequency in 62.8% (27/43), with parallel improvements in EEG findings and developmental outcomes. When used as a first-line therapy, KD achieves seizure reduction in approximately two-thirds of patients, with efficacy comparable to ACTH but with fewer side effects and relapses. ACTH, however, produces more rapid EEG normalization (43). For treatment-resistant WS/IS that does not respond to first-line therapies, KD has been shown to significantly improve seizure control, EEG outcomes, and overall quality of life, supporting its role as a second- or third-line option (45). In a prospective single-center study involving 104 WS/IS patients, spasms decreased by more than 50% in 64% of children at 6 months and in 77% at 1–2 years of KD treatment. Spasm cessation was achieved in 38 (37%) of patients within 2.4 months of KD initiation (46). Notably, the diet is especially effective in WS patients with identifiable pathogenic mutations, particularly those with *CDKL5* mutations (47). Among dietary variations, MAD has demonstrated comparable efficacy to CKD, with improved tolerability and higher compliance (48). Furthermore, MAD and CKD show similar effectiveness in seizure reduction and comparable rates of adverse effects such as vomiting, constipation, somnolence, and weight loss (49). Regarding KD initiation, non-fasting initiation such as a diet with a 3:1 ratio of fat to non-fat or ketogenic milk are also effective and better tolerated, with fewer adverse effects (50).

### 5.2 Ds

DS, or severe myoclonic epilepsy of infancy, typically manifests between 2 and 15 months of age, initially with febrile seizures followed by afebrile seizures of multiple types. The condition is highly sensitive to temperature and often leads to status epilepticus, developmental delay, and intellectual disability (51). Current pharmacologic options include sodium valproate and clobazam, often in combination with adjunctive agents such as cannabidiol, fenfluramine, topiramate, stavudine, levetiracetam, and clonazepam. However, efficacy remains suboptimal and side effects are considerable (52). KD has been shown to reduce seizures in approximately 50% of children with DS and is associated with rapid onset of action and good tolerability (53). A multicenter study confirmed the effectiveness of KD in drug-resistant DS, with a low incidence of adverse effects (54). Nevertheless, some studies report a decline in KD efficacy over time: seizure reduction ≥50% was observed in 63%, 60%, and 47% of patients at 3, 6, and 12 months, respectively. This may reflect declining adherence, treatment discontinuation, or a honeymoon effect (55). KD can reduce the frequency of various seizure types, including generalized, focal, atypical absence, and myoclonic seizures. Additionally, improvements in cognition, attention, and behavior have been reported in up to 75% of cases (56). Mutations in *SCN1A*, encoding the alpha subunit of the voltage-gated sodium channel, are present in 70%–80% of DS patients, and KD appears particularly effective in managing refractory seizures in this subgroup (56). Given its impact on myoclonic seizures and status epilepticus, as well as the enhanced compliance associated with liquid KD formulations in infants, early implementation of KD should be considered in DS management (57).

### 5.3 OS

OS, also known as early infantile epileptic encephalopathy, typically begins within the first 3 months of life. It presents with tonic or tonic-clonic seizures, often in clusters, and is characterized by burst-suppression patterns on EEG along with global developmental delay. The condition may later evolve into WS (58). First-line treatments include phenobarbital and phenytoin, while second-line agents may include midazolam, levetiracetam, and sodium valproate. Unfortunately, pharmacologic therapies are often ineffective, and the prognosis remains poor (59). Case reports describe successful seizure control with KD in children unresponsive to all known antiepileptic drugs. For example, a 5-year-old child exhibited significant seizure reduction and EEG improvement within 1 year of KD initiation (60). Similarly, a 1-month-old infant achieved seizure control following KD after failure of multiple agents including ACTH and intravenous immunoglobulin (61). While KD has shown promise in some epileptic encephalopathies, its effectiveness in OS remains underreported, necessitating larger clinical trials to validate its role.

### 5.4 LGS

LGS accounts for 2%–9% of pediatric epilepsy cases. It is characterized by tonic, atonic, and atypical absence seizures, along with generalized slow spike-and-wave activity on EEG and developmental or intellectual impairment (62). LGS is frequently drug-resistant, often secondary to conditions such as perinatal brain injury, intracranial infection, dysplasia, or metabolic disease. Treatment goals primarily focus on reducing seizure frequency and improving quality of life (62). First-line pharmacological agents include sodium valproate, lamotrigine, and topiramate, with other options including levetiracetam, clobazam, phenobarbital, and zonisamide. However, KD has emerged as an effective and well-tolerated alternative (62). In a cohort of 189 children with LGS, 47% achieved ≥50% seizure reduction after 3–36 months of KD therapy, with most responders showing improvements by 12 months (63). KD has also demonstrated efficacy in LGS associated with tyrosinemia type 1, mitochondrial disorders, and structural brain abnormalities, contributing to improved long-term outcomes (64–66). Furthermore, its efficacy has been reported to be comparable to vagus nerve stimulation in patients ineligible for surgical intervention (64). Common adverse effects include constipation, nausea, and vomiting, while serious events such as nephrolithiasis or osteoporosis are rare (65). Given the limited efficacy of pharmacologic treatments, early and sustained implementation of KD is recommended for LGS.

### 5.5 EMAS

EMAS, also known as Doose syndrome, is a rare generalized epilepsy that typically manifests between 7 months and 6 years of age. Seizure types include myoclonic-atonic, myoclonic, atonic, generalized tonic-clonic, absence, and tonic seizures, with EEG showing generalized spike-wave or polyspike-slow wave discharges. While development is typically normal before onset, many patients experience developmental stagnation or regression after seizures. Associated features may include tremor, ataxia, and scoliosis (66, 67). Corticosteroids, ethosuximide, and sodium valproate are effective in some cases, while levetiracetam and zonisamide offer additional seizure control. However, KD appears to be the most effective treatment currently available for EMAS (68). A multicenter retrospective study found that early KD initiation in drug-resistant EMAS led to sustained seizure control and better cognitive outcomes (69). Genetic studies suggest that *SLC2A1* mutations and glucose transporter 1 (GLUT1) deficiency may underlie some cases of EMAS, providing a mechanistic rationale for KD effectiveness (70). More than 50% of children treated with KD experienced ≥50% seizure reduction, and 18% achieved seizure freedom. These findings support early KD implementation in EMAS, rather than reserving it as a last resort (70).

### 5.6 DEE associated with other genetic mutations

DEEs exhibit marked genetic heterogeneity, necessitating genetic testing for precise diagnosis and treatment. Studies demonstrate favorable responses to the KD in patients with mutations in *SCN1A* (*n* = 18, responder rate = 77.8%, *p* = 0.001), *KCNQ2* (*n* = 6, responder rate=83.3%, *p* = 0.022), *STXBP1* (*n* = 4, responder rate = 100.0%, *p* = 0.01), and *SCN2A* (*n* = 3, responder rate = 100.0%, *p* = 0.041), with improvements in both seizure control and cognitive outcomes (9). In contrast, KD efficacy in *CDKL5-*related DEE remains inconsistent. While one small study reported no significant response (*n* = 10, responder rate = 0.0%, *p* = 0.031) (9), a larger registry-based study (*n* = 104) found that two-thirds (69/104) experienced changes in seizure activity after starting KD, with 88% (61/69) of these showing improvement; however, notably poor long-term efficacy persists (71). Case reports also suggest potential KD benefits in rare DEE subtypes associatedwith *ALG13* (72), *HCN2* (73), and *ATN1* (74) mutations, including seizure reduction and improved alertness. The current literature summarizing KD efficacy across major genotypes is presented in Table 1.

**Table 1:** Summary of KD effects in DEE with specific genetic mutations.

Gene	Sample size	Study type	Phenotypes	Response rate	Key benefits	References (Author of the article)
*SCN1A*	18	Cohort study	Early-onset epilepsy, fever-triggered seizures, cognitive decline	77.80%	Seizure reduction, cognitive improvement	Ko A
*KCNQ2*	6	Cohort study	Early infantile epileptic encephalopathy, tonic spasms	83.30%	Effective seizure control in Ohtahara syndrome	
*STXBP1*	4	Cohort study	Infantile-onset epilepsy, global developmental delay	100%	Seizure relief, EEG improvement	
*SCN2A*	3	Cohort study	Seizures starting in neonatal period, developmental regression	100%	Complete seizure control, ASM reduction	
*CDKL5*	10	Small-sample study	Early-onset seizures, Rett-like features, poor ASM response	0%	No significant effect observed	
*CDKL5*	104	Registry study	Same as above	88%	Short-term benefit, poor long-term efficacy	Lim Z
*ALG13*	1	Case report	Developmental delay, ID speech/visual impairment, infantile spasms	–	Seizure cessation, developmental milestone gains	Chand P
*HCN2*	1	Case report	Early-onset seizures, severe DD, abnormal EEG	–	>75% seizure reduction, normalized EEG	DiFrancesco JC
*ATN1*	1	Case report	Hypotonia, epilepsy, ID, digital anomalies, brain malformation	–	Sustained seizure control over 2 years	Xie Y

KD, ketogenic diet; DEE, developmental and epileptic encephalopathy; EEG, electroencephalogram; ASM, anti-seizure medication; CDG, congenital disorder of glycosylation; EIEE, early infantile epileptic encephalopathy; ID, intellectual disability; DD, developmental delay; CHEDDA, congenital hypotonia, epilepsy, developmental delay, digital anomalies.

## 6 Safety and tolerability of KD in DEE treatment

KD is generally considered a safe and well-tolerated treatment option for children with refractory epilepsy (75). It has been successfully implemented in adults and children, including infants as young as 6 weeks of age (76). Evidence suggests that children under 2 years old may represent the optimal population to initiate KD, especially those with intractable epilepsy of genetic origin (77). Currently, numerous studies support the efficacy of KD in the management of DEE. MAD and LGI are frequently employed alternatives to classical KD, demonstrating comparable seizure control with improved tolerability (78). In a 2018 study, Wirrell et al. assessed the effectiveness and safety of KD in infants under 12 months of age, showing response rates of 68%, 82%, and 91% at 1, 6, and 12 months of treatment, respectively, with seizure freedom achieved in 20%, 29%, and 27% of patients, respectively. Only two children experienced significant hypoglycemia at initiation due to vomiting and reduced oral intake; by 12 months, two patients had discontinued KD due to severe adverse reactions (79). Additional evidence supports the safety and efficacy of KD in neonates and infants under 3 months of age, with a low incidence of both acute and delayed adverse reactions (80). In addition, case studies have shown that KD is well tolerated and safe in premature infants, with transient and asymptomatic hypoglycemia and weight loss resolved (81).

Common early adverse effects of KD include hypoglycemia and dehydration, typically related to fasting and reduced caloric intake. Short-term gastrointestinal side effects such as vomiting, constipation, and diarrhea are frequently reported (82). Hyperlipidemia is another common complication, affecting approximately three-quarters of children on KD. Long-term use of KD may result in metabolic disturbances including hypercholesterolemia, hypertriglyceridemia, hyperuricemia, hepatic dysfunction, electrolyte imbalances, atherosclerosis, decreased bone mineral density, cardiomyopathy, nephrolithiasis, optic neuropathy, anemia, and deficiencies in vitamins and minerals; pancreatitis is rare but has been reported (83, 84). While these adverse effects are relatively uncommon, strict KD regimens may impair growth and development due to limited protein and caloric intake (85). This may also relate to reduced calcium and vitamin D levels during treatment, contributing to osteopenia (86). Given the potential for cardiovascular, renal, skeletal, and growth-related complications with prolonged KD use, patients should undergo regular monitoring, and appropriate interventions should be employed to mitigate these risks.

## 7 Outlook

For many children with DEE who suffer from catastrophic seizures, the therapeutic goal often shifts from complete seizure control to seizure reduction and improvement in cognition and quality of life. KD is commonly introduced after the failure of multiple antiseizure medications and even epilepsy surgery. It is considered a first-line treatment for metabolic epilepsies such as GLUT1 deficiency syndrome and pyruvate dehydrogenase deficiency (PDHD), and it remains an established treatment option for DEE. In drug-resistant DEE cases, KD may be considered early, except in patients with absolute contraindications such as porphyria, β-oxidation defects, or primary carnitine deficiency. KD not only reduces or controls seizures in most DEE cases, but also improves electroencephalographic findings, behavior, cognition, and alertness. Prolonged treatment duration is associated with more pronounced benefits.

However, KD is contraindicated in certain metabolic disorders, including fatty acid oxidation defects (e.g., CPT deficiency), pyruvate carboxylase deficiency, porphyria, mitochondrial respiratory chain disorders, and severe hepatic or pancreatic dysfunction, due to the risk of life-threatening metabolic crises (87). Though gastrointestinal symptoms such as nausea, vomiting, and diarrhea are the most common adverse effects, serious complications are rare. Other side effects, such as nephrolithiasis, hyperlipidemia, and micronutrient deficiencies, can often be corrected through dietary modification and supplementation (88). Therefore, close clinical monitoring, timely adjustments to the KD regimen, and proactive prevention of adverse effects are essential for safe implementation (89).

KD is effective across various seizure types and is generally suitable for most children with DEE. Treatment regimens should be tailored according to patient age and clinical characteristics. Although KD is a safe and effective therapy for DEE, its impact on gut health warrants further investigation, and formulation improvements are needed to enhance palatability and adherence. Genetic testing facilitates early and accurate diagnosis of DEE subtypes, enabling the development of personalized ketogenic interventions. Targeted nutritional strategies may prove less restrictive, more efficacious, and associated with fewer adverse effects. Due to limitations in sample size and follow-up duration, further studies are required to evaluate the long-term safety and efficacy of KD. In conclusion, well-designed, long-term clinical trials are needed to establish personalized, safer, and more sustainable dietary therapies for children with DEE.
